# Tribological Behaviour of Mg–3Zn–xZr–1Y Alloys for Biomedical Implant Applications

**DOI:** 10.3390/ma19214452

**Published:** 2026-10-22

**Authors:** Camryn Brown, Mohsen Feyzi, Mahdi Jalilvand, Win K. Look, Masataka Ijiri, Wenlong Xiao, Reza Hashemi

**Affiliations:** 1 College of Science and Engineering, Flinders University, Tonsley, SA 5042, Australia; 2 Department of Mechanical Systems Engineering, Tokyo Metropolitan University, Tokyo 192-0065, Japan; ijiri@tmu.ac.jp; 3 School of Materials Science and Engineering, Beihang University, Beijing 100191, China

**Keywords:** magnesium alloys, tribology, wear, biomedical implants

## Abstract

Magnesium (Mg) alloys are promising biodegradable materials for orthopaedic implants because of their bone-like mechanical properties and biocompatibility. However, their clinical application is limited by rapid degradation, caused by the combined effects of wear and corrosion in physiological environments. Understanding the tribological behaviour of these alloys is essential for improving both the design and performance of medical implants. This paper studies recently developed Mg–3Zn–xZr–1Y alloys with a focus on the influence of zirconium (Zr) additions (0.25–1.00 wt%) on the tribological performance for biomedical applications. The developed alloys were tested under dry and physiological conditions simulated by phosphate-buffered saline (PBS) using a customised reciprocating ball-on-disc tribometer. The normal loads of 6, 17.5 and 30.8 N were selected to mimic a range of typical contact pressures experienced by relevant medical implants. The surface damage of the tested alloys was characterised using scanning electron microscopy (SEM) and energy-dispersive X-ray spectroscopy (EDS), and material loss was determined by confocal microscopy. Volume loss analyses showed that PBS significantly reduced material loss as a result of the lubrication effect and the possible formation of tribochemical films. The effect of Zr content on the material loss was found to be highly coupled with the effect of both normal load and the testing environment. SEM characterisations showed that abrasive wear, primarily ploughing and localised cutting, was the dominant degradation mechanism under dry conditions; whereas, in PBS conditions, the dominant degradation mechanism was found to change from abrasive wear to a combination of moderated abrasive wear and oxidation/corrosion.

## 1. Introduction

Recently, there has been a significant increase in the investigation of magnesium (Mg) alloys for biomedical applications, particularly in orthopaedic and cardiovascular fields. They have emerged as prime candidates for metallic implants due to their biodegradability, biocompatibility, and favourable mechanical properties that are close to natural bone [1]. The biodegradability of Mg alloys allows them to safely degrade within the human body upon tissue healing [2]. This characteristic reduces the need for secondary removal surgeries and promotes natural healing. Unlike other common metallic biomaterials, such as titanium alloys and stainless steels, which live permanently in the body, Mg-based implants can dissolve gradually in physiological environments [2].

Mg alloy bone-like qualities are presented by their low density, comparable Young’s modulus, and high stiffness-to-mass ratio [3]. Therefore, they have inherent advantages in mitigating the stress shielding effect, with excellent specific strength and specific stiffness [1]. Typical uses for Mg alloys can be seen in a variety of surgical applications due to their ability to improve osteointegration and enhance the stability of the implant [2]. Therefore, there is promising development of Mg alloys in orthopaedic and cardiovascular implants, urology, neural applications, respirology, surgical wires and staplers, and antitumor activities [2]. Despite the benefits that Mg alloys offer, their clinical translation remains limited in biomedical applications due to their rapid and non-uniform corrosion rate in physiological environments. Due to the chloride-rich physiological environment, Mg alloys have been reported to often experience stress corrosion cracking (SCC) under tensile stresses [4]. Their poor corrosion resistance can result in loss of mechanical integrity with the release of magnesium ions and hydrogen gas to the surrounding tissues [5]. Their non-toxic byproducts can be excreted through human metabolism, and a build-up of hydrogen can cause tissue layers to separate and potentially damage cells [5,6].

While the corrosion behaviour of Mg alloys in the body environment has been a major research focus in the literature [2,4,7,8], some medical devices and implants also experience combined wear and corrosion, known as tribocorrosion [9,10,11]. Clinical implementation is challenged by tribocorrosion, as mechanical wear exacerbates electrochemical degradation in a chloride-rich physiological environment [12]. This phenomenon can largely occur at bone fixation sites [13,14,15,16], where degradation mechanisms include mechanically assisted corrosion on fixation screws [14]. Tribocorrosion accelerates material loss through the relationship between electrochemical degradation and mechanical abrasion [17,18,19]. This process can negatively impact implant longevity and biocompatibility [20].

There are very few studies focusing on the tribology and tribocorrosion behaviour of Mg alloys for biomedical applications in which normal contact stresses are applied to Mg alloy samples to produce wear depths on the surface [21,22]. Hu [21] found that the surface area of Mg alloy AZ31B and the depth of the wear marks gradually increase with the increased normal load of 20, 40, and 60 N. The respective volume loss was reported as 0.83, 2.52, and 3.58 $\times 10^{6}\;\mu m^{3}$ [21]. Furthermore, Megret et al. [22] discussed delamination of the oxide layer observed in the middle of the wear tracks after their tribocorrosion testing on Mg alloy AZ31. As such, 0.008, 0.012, and 0.05 mm^3^ of worn volume were calculated under the normal forces of 2, 5, and 8 N, respectively [22]. Previous results show that an increased normal load enhances the tangential stiffness of the Mg alloy and inhibits sliding [21]. Also, the increased tangential force leads to plastic deformation of the material, which increases wear volume and depth [21].

Improved wear-resistance properties can be achieved through metallurgical modification and surface modification for Mg alloys [5]. Metallurgical modification optimises compositions and the microstructure by fabrication and heat treatment [5]. Surface modification methods improve performance by preparing protective coatings or changing the surface features of materials [5]. In the literature, surface modification is the primary strategy used to mitigate tribocorrosion in Mg alloys [5,23,24]. Such approaches can be broadly divided into conversion coatings, which chemically modify the substrate surface, and non-conversion coatings, which deposit an external barrier layer [16]. However, these coatings have significant limitations, with the most notable being the potential for delamination, which may lead to adverse effects in the body.

There have been efforts to develop new Mg alloys that can offer enhanced corrosion properties for biomedical applications [25,26,27,28]. In a recent study [25], Mg-xZn-yZr-1Y alloys were developed with variations in Zn (1, 3, 5 wt%) and Zr (0.25, 0.5, 0.75, 1.00 wt%) to enhance the corrosion resistance in a phosphate-buffered saline (PBS) solution to mimic physiological conditions. This research highlighted that the rapid corrosion rate significantly decreased due to the lower magnitudes of corrosion current density and more positive open-circuit potential (OCP), indicating resistance to corrosion initiation and propagation [25]. These findings confirmed Zr’s crucial role in improving surface stability and integrity in physiological conditions [25]. Therefore, the next logical step would be to understand how these new alloys’ microstructural and corrosion improvements could possibly influence the tribological behaviour of the developed alloys.

Despite significant progress in corrosion studies of new Mg alloys, the tribology mechanisms of Zr-modified Mg alloys under physiological loading are not fully studied yet; therefore, the exploration of the relationship between mechanical wear and electrochemical degradation in Zr-modified Mg alloys is necessary to advance their future use in biomedical applications. This paper therefore aims to evaluate the tribological behaviour of new Mg alloys with varying Zr contents (0.25–1.00 wt%) under simulated physiological environments and loading conditions. It aims to establish the desirable Zr concentration(s) for balancing wear resistance, corrosion stability, and mechanical integrity in biomedical applications.

## 2. Materials and Methods

### 2.1. Materials and Sample Preparation

Four Mg alloys, including Alloy 1 (Mg–3Zn–0.25Zr–1Y), Alloy 2 (Mg–3Zn–0.50Zr–1Y), Alloy 3 (Mg–3Zn–0.75Zr–1Y), and Alloy 4 (Mg–3Zn–1.00Zr–1Y), were synthesised using high-purity Mg (99.99 wt%), Zn (99.99 wt%), Mg–30%Zr, and Mg–30%Y master alloys. The alloy preparation commenced by melting pure Mg in a resistance furnace at approximately 720 °C under a protective atmosphere consisting of N_2_ and SF_6_ (99:1). After the magnesium had fully melted, pure Zn, together with the required master alloys, was introduced, and the melt temperature was increased to 750 °C. The molten alloy was mechanically stirred at 300 rpm for 3 min to promote chemical homogeneity, followed by a 20 min holding period at 720 °C. Finally, the melt was cast into a steel mould preheated to 200 °C, producing cylindrical Mg alloy ingots with dimensions of 60 mm in diameter and 150 mm in length.

The ingots were sectioned and cut into a series of rectangular samples measuring 18 mm × 18 mm × 5 mm using a precision saw. These sample dimensions were finalised based on the maximum length that the tribotester’s chamber can hold where the sample sits. All samples underwent the same manual polishing procedure using 80-, 400-, 800-, 1000-, and 2000-grit sandpapers to ensure a consistent and comparable initial surface condition prior to tribological testing. This ensured that the resulting surface finish was representative of the surface conditions typically encountered in machined medical devices [29]. The process was carried out manually to remove contaminants, producing a smooth and uniform surface for a consistent baseline.

**Figure 1:**
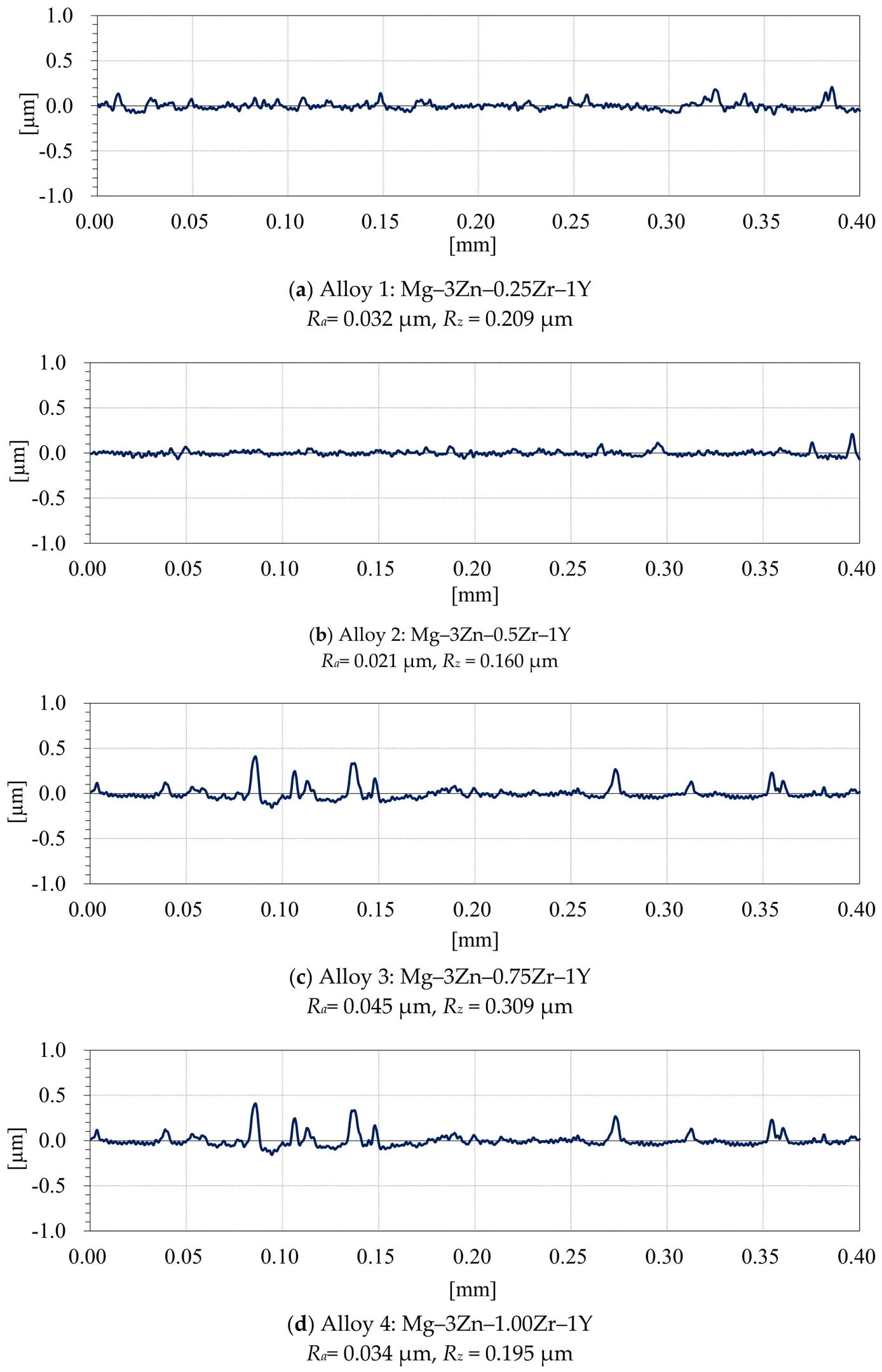
Average surface profile and the corresponding average surface roughness parameters for various Mg alloys.

Following cleaning with methanol, surface roughness measurements, including line-scan profiles, were performed on all four Mg alloys using a surface roughness tester (Mitutoyo; Model: Surftest SJ-410, Kawasaki, Japan). For each alloy, measurements were taken at three different locations on the surfaces of three separate samples. The average surface profile and the corresponding average surface roughness parameters, namely the arithmetic mean roughness (*R_a_*) and maximum height of the roughness profile (*R_z_*), were then determined for each alloy, as shown in Figure 1.

### 2.2. Tribology Experiments

**Figure 2:**
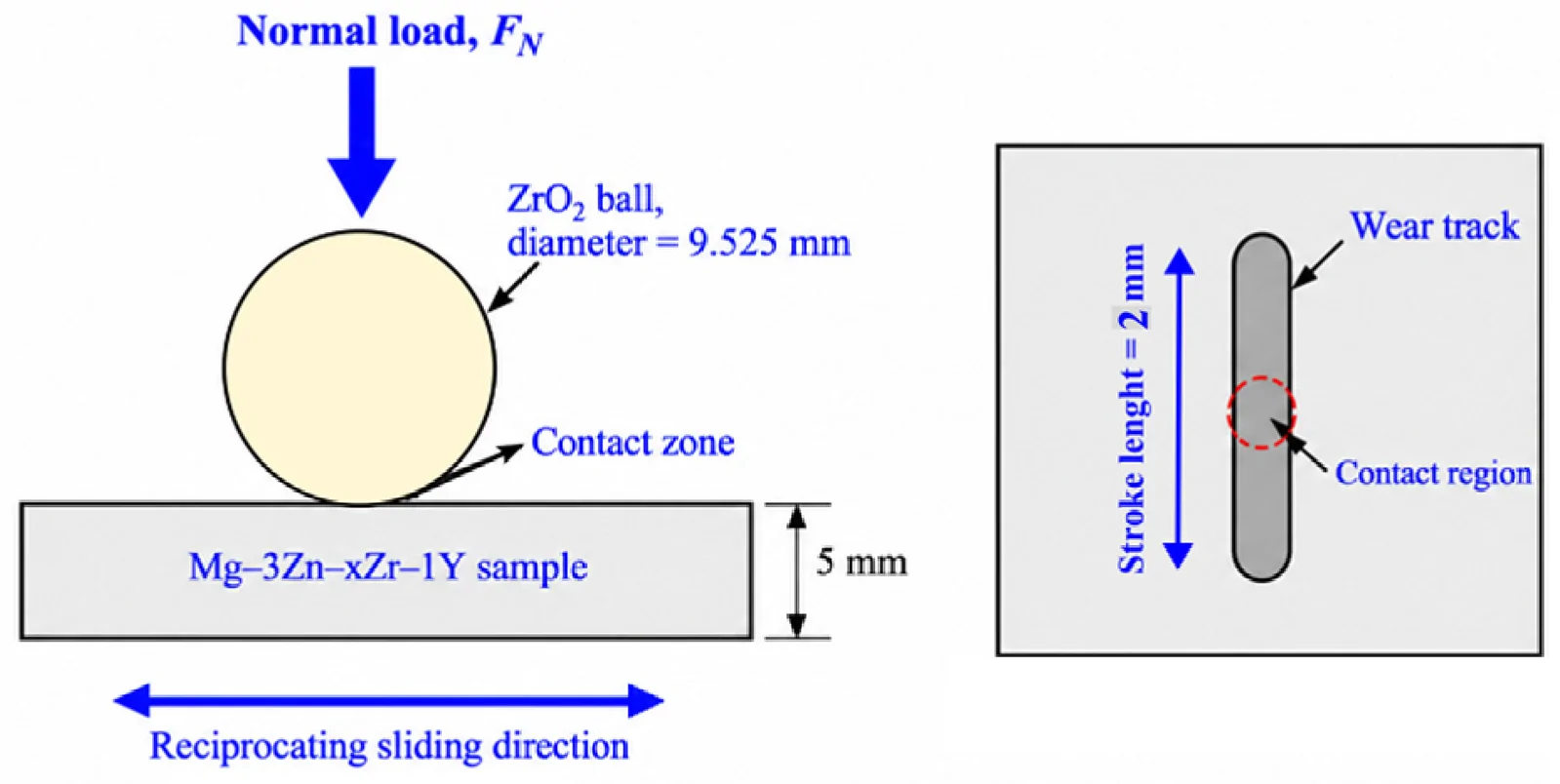
Simplified schematic of geometry and configuration of tribological contact.

A custom-built ball-on-disc tribology testing apparatus was utilised to test the Mg alloy samples in both dry and physiological conditions [29,30]. Zirconia balls (ZrO_2_ > 96%) (Shandong Ultraming Fine Ceramics, Zibo, China) with a diameter of 9.525 mm were used for the experiments (Figure 2).

The sliding motion on the disc (Mg alloy samples) was generated by an electromagnetic actuator (LinMot; Model: P10-70  ×  400, Lake Geneva, WI, USA). This motion was directly transmitted to the chamber through the horizontal sliding stage that was firmly connected. A phosphate-buffered saline (PBS) solution (Sigma-Aldrich, St. Louis, MO, USA) with a pH of 7.4 was added to the chamber to mimic physiological conditions for the tribology tests. A polymeric insert was developed to firmly house the sample within the chamber. The whole perimeter of the rectangular sample was carefully sealed with silicone adhesive to prevent any possible leakage of the PBS solution. The sample surface was then cleaned with methanol to eliminate any possible contamination in the preparation and/or seating steps. The ball gripper that houses the zirconia ball was gently lowered to make initial contact with the disc surface, and the amounts of dead weight were then adjusted to achieve a specific level of normal force. The normal forces of 6, 17.5, and 30.8 N were chosen to ensure that the oxide layer was removed from the interface. This resulted in maximum Hertzian contact pressures of approximately 421, 601, and 735 MPa. The increments in the normal force magnitudes were selected such that a reasonably wide range was covered for exploring the volume loss trend vs. normal force. The actuator was active to apply a nominal sliding distance of 2 mm at a frequency of 1 Hz for 1800 cycles. A selected number of experiments were repeated three times to ensure reproducibility. Table 1 summarises the investigated materials, their nominal chemical composition, testing parameters and conditions, and applied normal loads.

### 2.3. Surface Damage Characterisations

Following completion of the tribology tests, the tested Mg alloy samples were ultrasonically cleaned in ethanol and examined using a field-emission scanning electron microscope (SEM) (FEI Inspect; Model: F50, Hillsboro, OR, USA) equipped with an energy-dispersive X-ray spectroscopy detector (EDS). The SEM examination focused on the morphology of the wear scars, and EDS point analyses were conducted at selected locations to determine the local elemental composition of the examined surfaces. For the samples tested in PBS solution, the regions adjacent to the wear scars were also examined. Imaging was conducted at an accelerating voltage of 20 kV, a working distance of approximately 10 mm, and an instrument spot-size setting of 3 nm. A total of six samples tested under a normal force of 30.8 N were selected for SEM and EDS characterisation. Under dry sliding conditions, all four alloys were examined. Under PBS conditions, the Mg–3Zn–0.25Zr–1Y (Alloy 1) and Mg–3Zn–1Zr–1Y (Alloy 4) alloys were selected to represent the lowest and highest Zr contents, respectively.

### 2.4. Volume Loss Estimation

To determine volume loss in every sample, a confocal microscope (Olympus Lext OLS5000, Tokyo, Japan) was used. The confocal microscope captured optical images that allowed for precise three-dimensional reconstruction of the worn topography in the region of interest (ROI), i.e., the wear-damaged area on the material surface. Images were taken with an objective lens of Mplapon 20× and 3D fine colour scanning mode of the samples tested in the dry condition. Comparatively, an objective lens of Mplapon 20× and laser fine scanning mode were used for the samples submerged in PBS solution. The change in the scanning mode for the samples submerged in PBS was due to the shallower depth of the wear damage. A line scan was completed on all samples to capture the worn profile (along each scar’s width at its mid-length). To ensure the reliability and repeatability of the volume loss measurements, the volume loss measurement was carried out for all samples.

## 3. Results

### 3.1. Surface Characterisations

#### SEM & EDS

**Figure 3:**
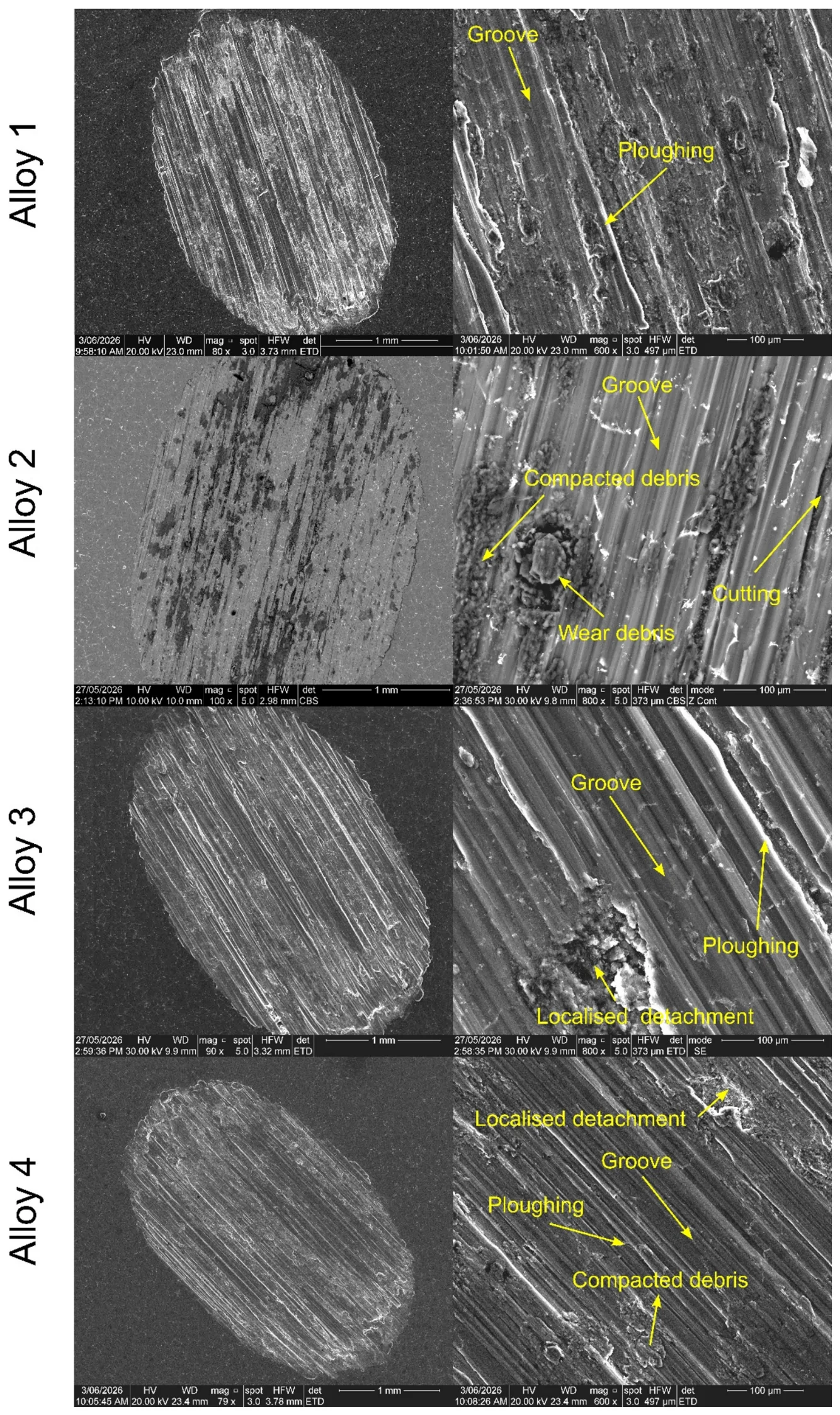
SEM micrographs of the wear scars and selected regions of interest for samples tested under a normal force of 30.8 N in dry conditions. From top to bottom: Alloy 1 (Mg–3Zn–0.25Zr–1Y), Alloy 2 (Mg–3Zn–0.50Zr–1Y), Alloy 3 (Mg–3Zn–0.75Zr–1Y), and Alloy 4 (Mg–3Zn–1.00Zr–1Y).

Figure 3 presents low- and high-magnification SEM micrographs of the wear scars formed on the Mg alloys under a normal force of 30.8 N in dry conditions. All samples exhibited numerous continuous grooves and scratches aligned with the sliding direction. At higher magnification, plastically displaced material and raised ridges were observed along the sides of several grooves, indicating pronounced ploughing of the relatively soft Mg matrix. Narrower and more sharply defined scratches were also present, suggesting that localised cutting contributed to material removal. In addition, localised surface flaking and accumulations of wear debris were observed within several wear scars. Comparing the alloys with the lowest and highest Zr contents, Alloy 1 (0.25Zr) exhibited relatively coarse and irregular grooves, together with more pronounced ploughing and plastic displacement of the Mg matrix (Figure 2). In contrast, Alloy 4 (1.00Zr) showed finer and more uniformly distributed grooves with less extensive surface deformation, although localised material delamination and debris-rich regions were still observed.

**Figure 4:**
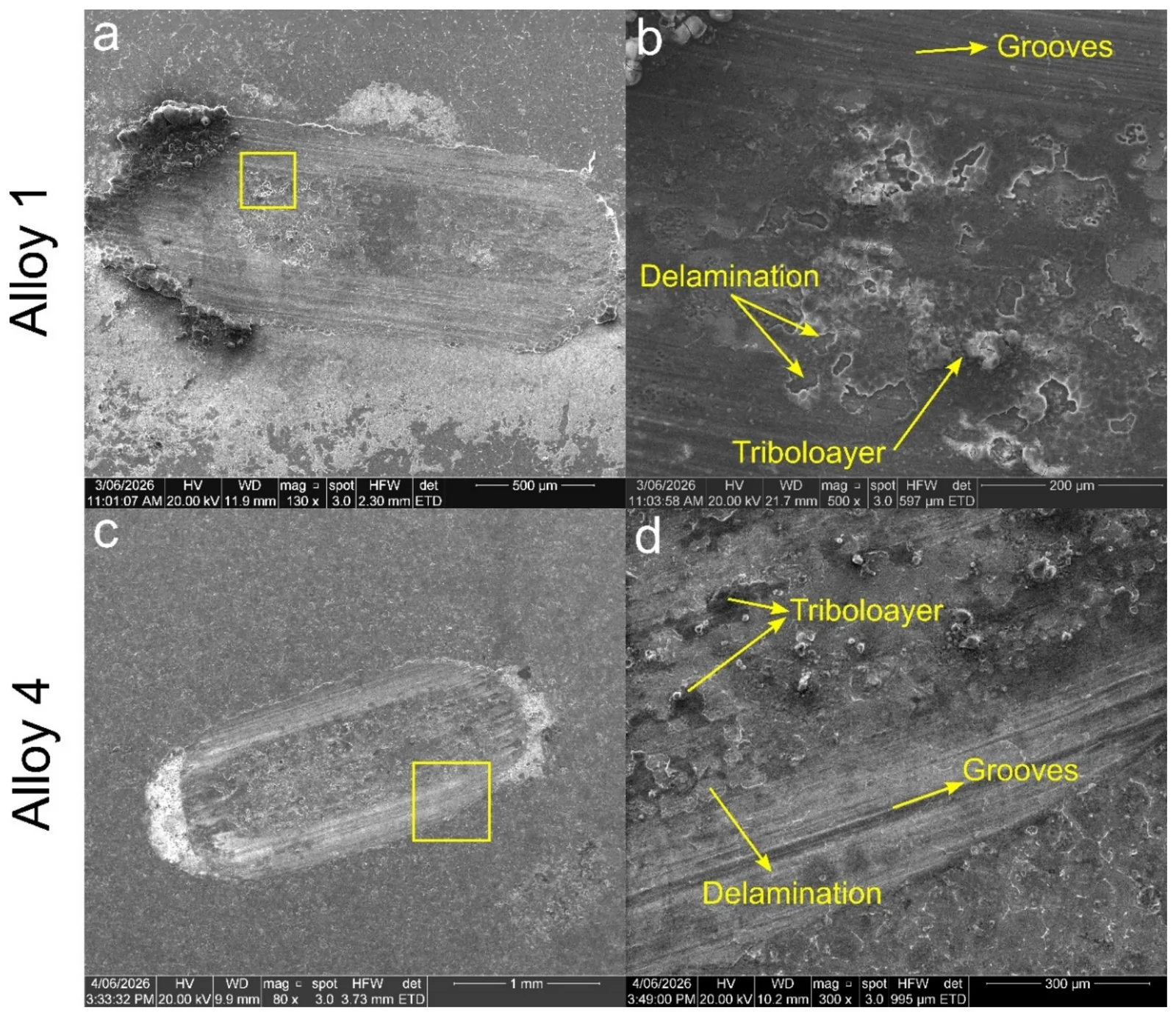
SEM micrographs of the wear scar tested under a normal force of 30.8 N in PBS solution for Alloy 1: (**a**) overall morphology of the wear scar and (**b**) enlarged view of the marked region; Alloy 4: (**c**) overall morphology of the wear scar and (**d**) enlarged view of the marked region.

Figure 4 presents SEM images of the wear scars formed on Alloy 1 and Alloy 4 during sliding in PBS solution under a normal force of 30.8 N. At low magnification (Figure 4a–c), both alloys exhibited shallow grooves and ploughing marks aligned with the sliding direction, together with localised material accumulation and plastic displacement along the scar boundary at some locations. Higher-magnification images of the boxed regions (Figure 4b,d) revealed that portions of the wear scars were covered by a discontinuous and heterogeneous tribologically modified layer, hereafter referred to as the tribolayer. Longitudinal grooves remained visible across and between regions of the tribolayer, indicating the continued occurrence of abrasive wear. The tribolayer exhibited local cracking, fragmentation, and delamination, resulting in irregular exposed regions within the wear scars. These features indicate repeated formation, deformation, and removal of the tribolayer during cyclic sliding. Overall, the observed morphology indicates that wear in PBS involved a combination of ploughing, tribolayer formation, and localised tribolayer delamination. Compared with the samples tested under dry conditions, the grooves were less sharply defined, and fewer cutting marks were observed, indicating a reduction in the severity of abrasive wear in PBS. The worn-surface morphologies of Alloy 1 and Alloy 4 were similar to some extent, with both exhibiting comparable features, and no substantial morphological differences between the two alloys under PBS conditions.

The EDS point analysis results, averaged from various selected locations within the wear scar of Alloy 1 (0.25Zr) tested under dry conditions, showed the predominant content of magnesium (91.49 wt%) along with 2.14 wt% Zn, 0.78 wt% O, and 5.46 wt% C. For Alloy 4 (1:00Zr), the EDS measurements resulted in Mg (97 wt%), Zn (2.05 wt%), O (1.75 wt%), and C (0.15 wt%). The low oxygen content indicates that substantial oxidation and the formation of an oxygen-rich tribologically modified layer did not occur under dry sliding. The carbon content detected in both alloys may be associated with local surface contamination. Overall, both alloys exhibited broadly similar Mg-dominated elemental compositions with low oxygen content, indicating that increasing the Zr content from 0.25 to 1.00 wt% did not produce a substantial difference in the chemistry of the analysed worn surfaces under dry conditions.

**Figure 5:**
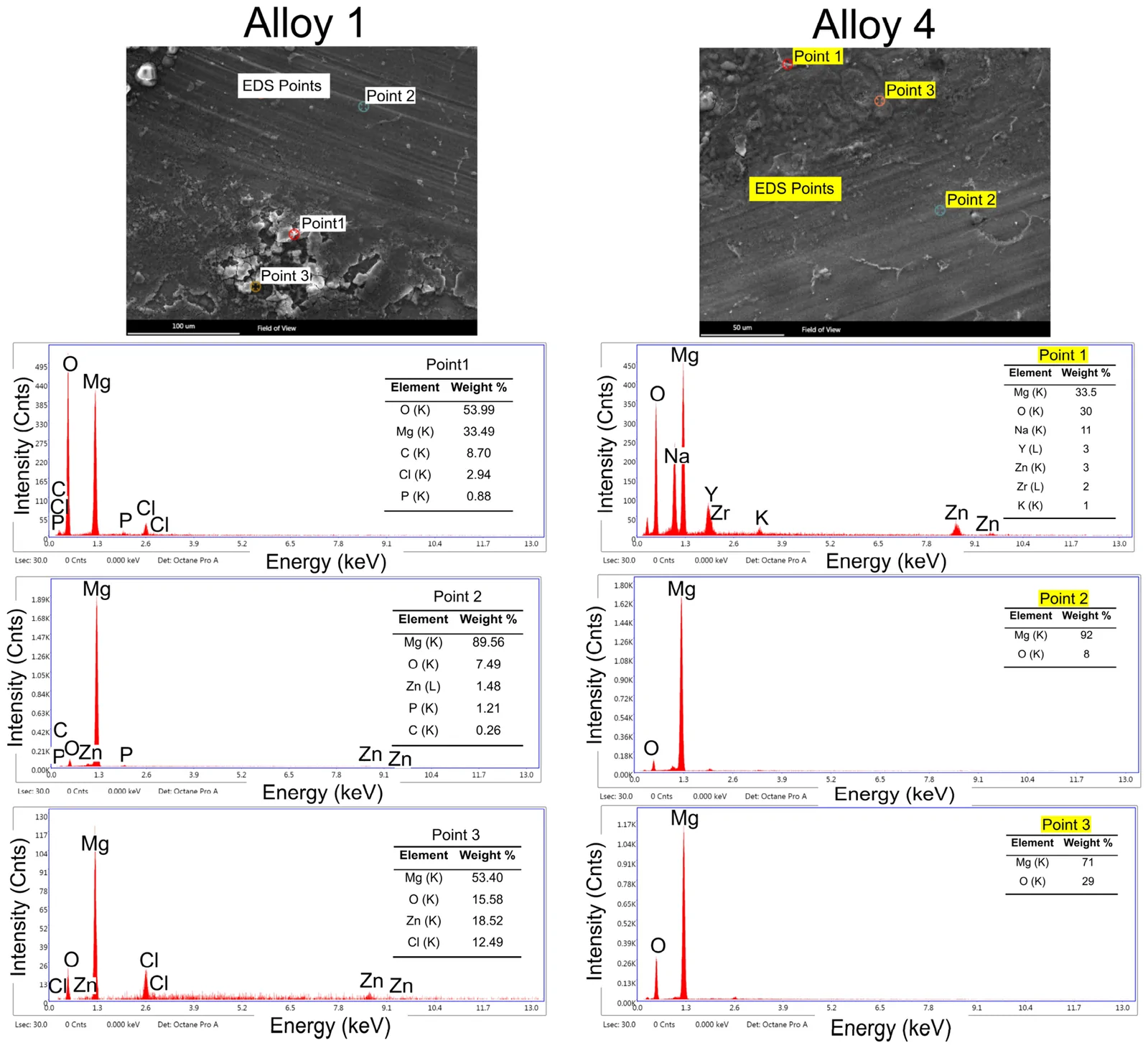
EDS point analysis results obtained from selected locations within the wear scars of Alloy 1 and Alloy 4 tested in PBS conditions.

As illustrated in Figure 5, EDS point analyses conducted at selected locations within the wear scars of Alloy 1 (0.25Zr) and Alloy 4 (1Zr) tested in PBS revealed clear compositional differences between the tribologically modified regions and the comparatively smoother grooved areas. For Alloy 1, Point 1, located within the delaminated tribolayer, contained a high O content of 53.99 wt%, together with 33.49 wt% Mg and minor amounts of C, Cl, and P. Point 3, located within another tribolayer region, also showed an elevated O content of 15.58 wt%, together with Mg, Zn, and Cl. In contrast, Point 2, located within the smoother grooved region, was predominantly Mg (89.56 wt%) and contained only 7.49 wt% O. A similar trend was observed for Alloy 4, where Point 1 within the tribolayer contained 30 wt% O, together with Mg and smaller amounts of Na, Y, Zn, Zr, and K, while Point 3 contained 29 wt% O and 71 wt% Mg. In comparison, Point 2, within the grooved region, was predominantly Mg (92 wt%), with only 8 wt% O. The substantially higher oxygen content within the tribolayer regions of both alloys indicates pronounced surface oxidation during sliding in PBS, while the detection of electrolyte-derived elements such as Na, K, Cl, and P further indicates interaction of the worn surface with the PBS solution. Overall, both alloys exhibited a similar compositional trend, characterised by an oxygen-rich tribologically modified layer surrounded by less oxidised, Mg-rich grooved regions, suggesting that increasing the Zr content from 0.25 to 1.00 wt% did not substantially alter the general chemistry of the worn surface under PBS conditions.

### 3.2. Surface Profilometry

**Figure 6:**
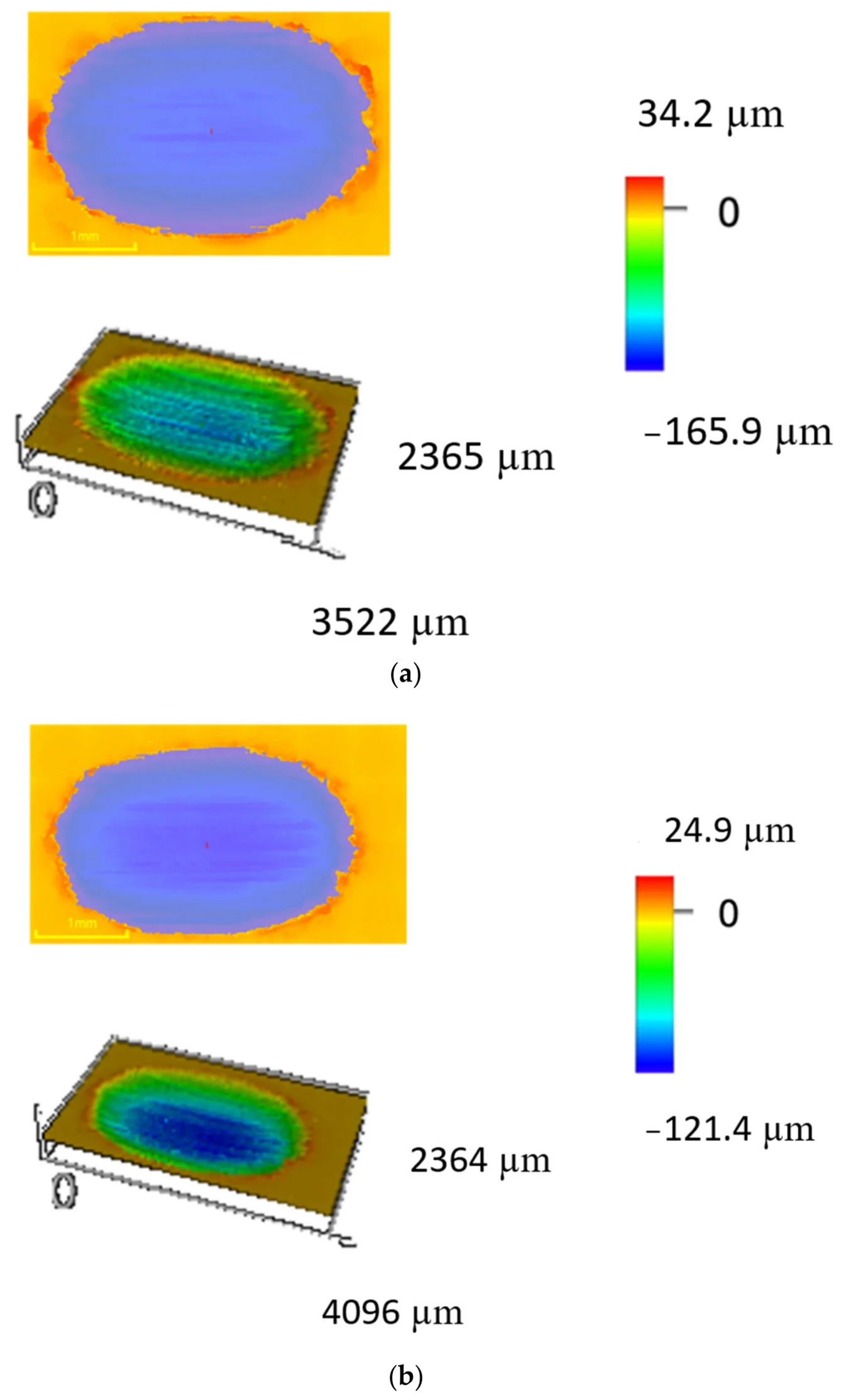

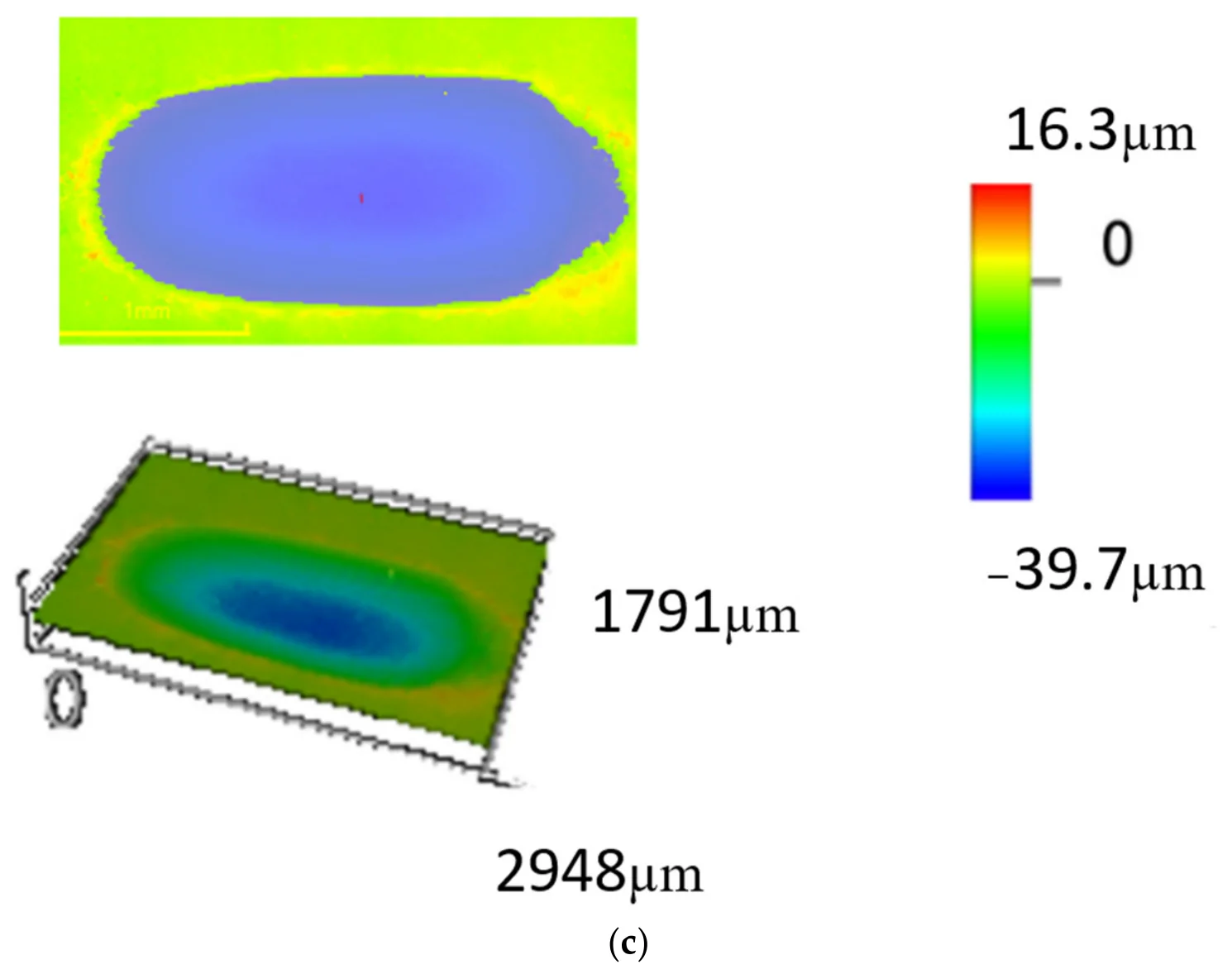
Three -dimensional wear morphology of (**a**) 0.25Zr alloy tested in dry condition, (**b**) 1.00Zr alloy tested in dry condition, and (**c**) 1.00Zr alloy tested in PBS.

**Figure 7:**
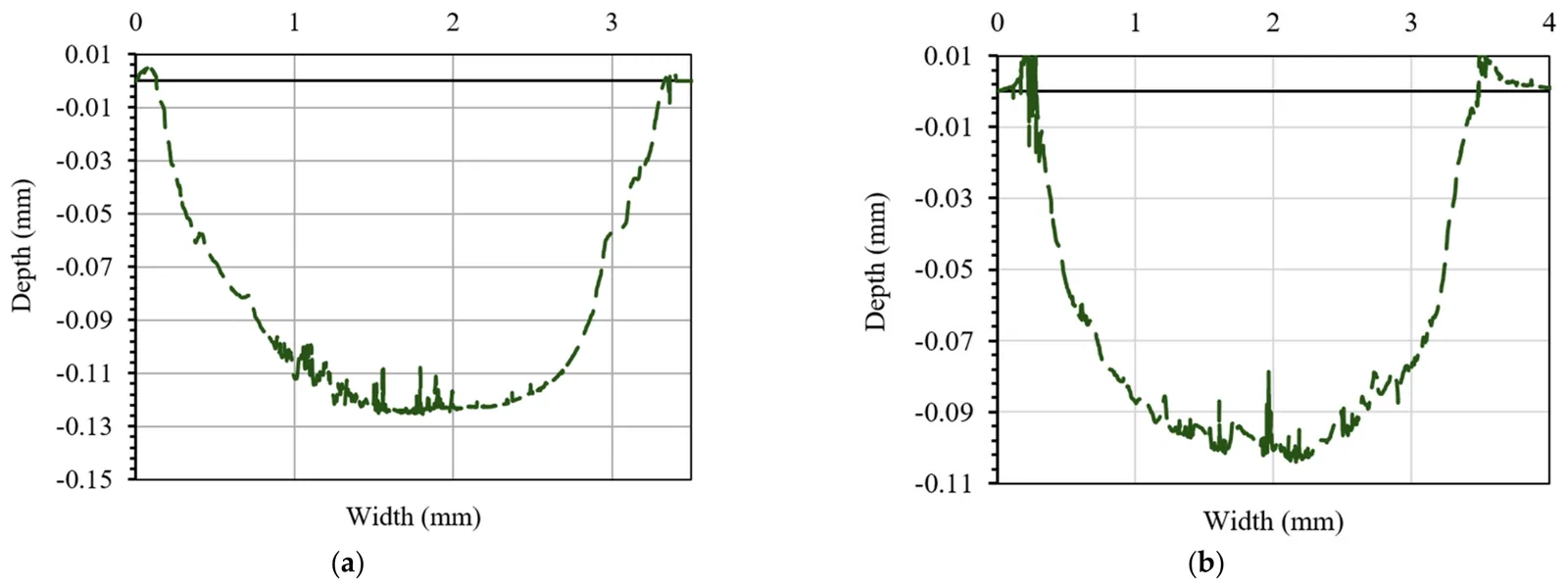

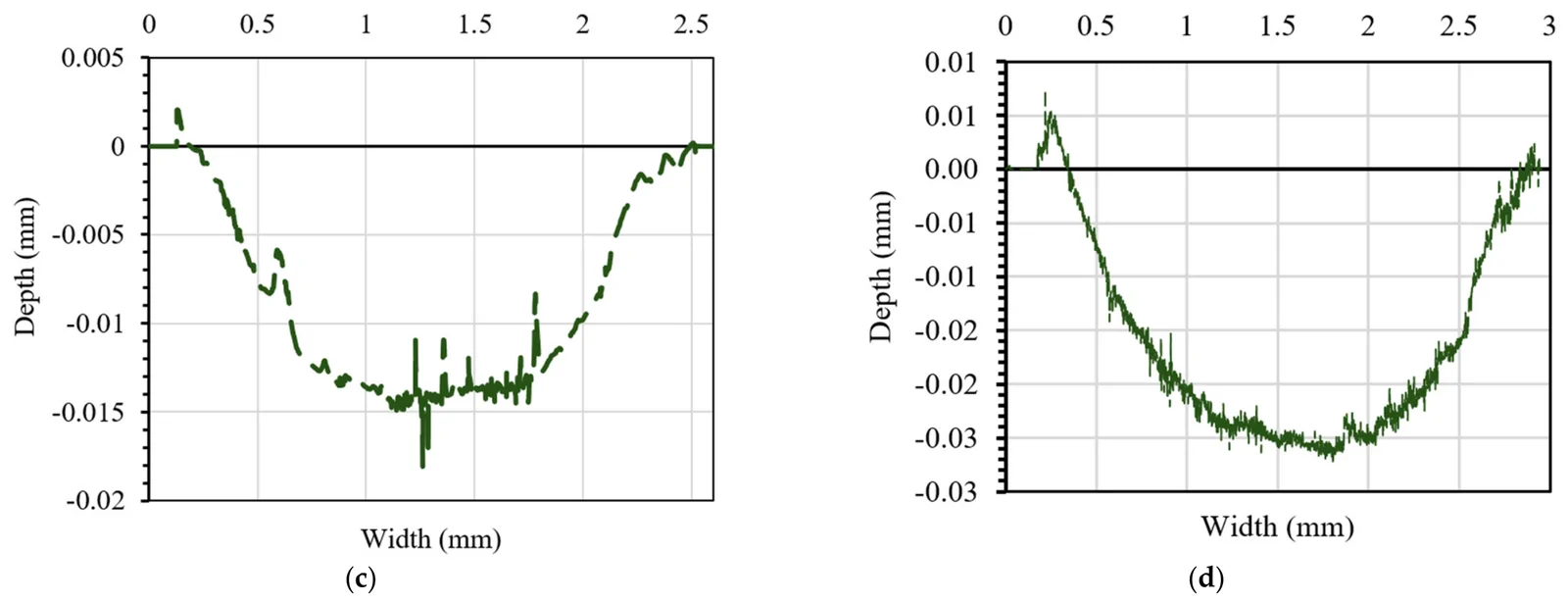
The cross-sectional wear profile of worn surfaces of different alloys tested under a normal load of 30.8 N: (**a**) 0.25Zr alloy tested in dry condition, (**b**) 1.00Zr alloy tested in dry condition, (**c**) 0.25Zr alloy tested in PBS, and (**d**) 1.00Zr alloy tested in PBS.

The three-dimensional wear morphologies obtained using confocal microscopy were used to estimate the volume loss from all the samples. The measurements from the three repetitions for the selected samples were in good agreement, with differences of less than 10%, indicating good repeatability of the volume loss measurements. Figure 6 presents the wear morphology of selected samples of various alloys tested under a normal load of 30.8 N. Also, the average wear profile from three line scans (along each scar’s width at its mid-length) is shown in Figure 7 for the alloys containing the highest and lowest Zr concentrations tested in both environments under a normal load of 30.8 N.

Clear differences in wear scar geometry are evident between the dry and PBS environments. Under dry conditions, both the 0.25 and 1.00Zr alloys exhibited deeper and well-defined wear scars with steep sidewalls (Figure 7a,b). The 0.25Zr alloy experienced the greatest wear depth of around 166 µm below the original surface, whereas the 1.00Zr alloy showed a shallower wear profile with a maximum depth of around 121 µm. These deep profiles are consistent with the severe wear mechanisms observed in the SEM images, where cutting and ploughing produced substantial material removal in dry conditions. In contrast, the surface profiles obtained after testing in PBS were considerably shallower for both alloys. The 0.25Zr alloy exhibited only a maximum depth of approximately 15 μm (Figure 7c), and the maximum wear depth of the 1.00Zr alloy was approximately 39 μm (Figure 7d). These two depths show a clear reduction in the wear intensity within the PBS environment compared to that in the dry conditions. Overall, the width of the wear profiles in the dry condition is greater than that in PBS; however, this width does not change noticeably with the changes in Zr content in each environment (Figure 7a–d). For instance, in dry conditions, the wear scar width was ~3.28 mm and ~3.2 mm for 0.25Zr and 1.00Zr alloys, respectively, which shows an approximate 3% difference. These results were consistent with the volume loss results, in which the volume loss could be reduced in the PBS environment, particularly for the 1.00Zr alloy (compared to the 0.25Zr alloy).

### 3.3. Volume Loss

In this study, three variables could affect the results of the volume loss. These three variables included the percentage of the Zr content across different alloys, the normal load applied to each alloy across the two environments, and the environmental conditions. The influence of these parameters on the volume loss is detailed below.

**Influence of Zr content**

**Figure 8:**
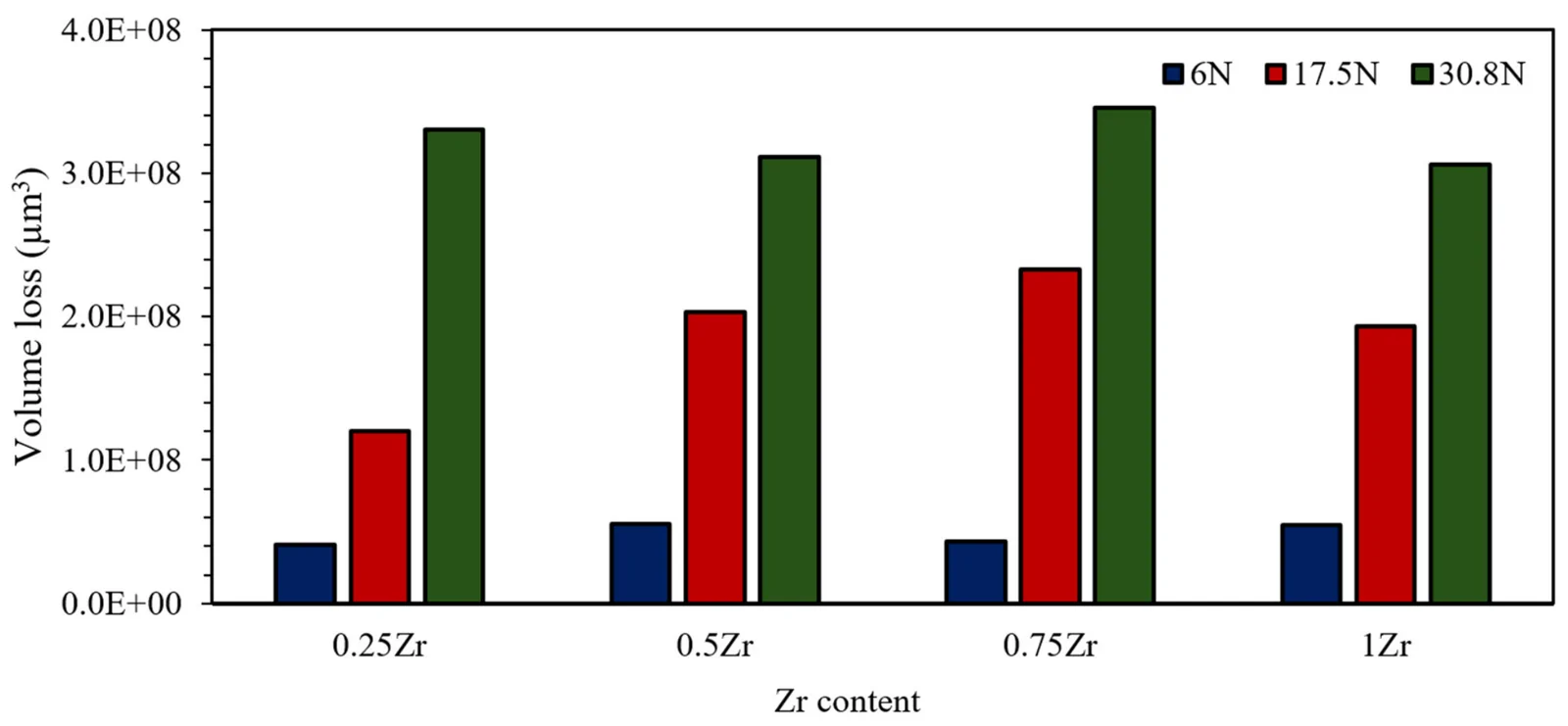
Volume loss of various Mg alloys in dry conditions under the three normal loads of 6, 17.5, and 30.8 N.

**Figure 9:**
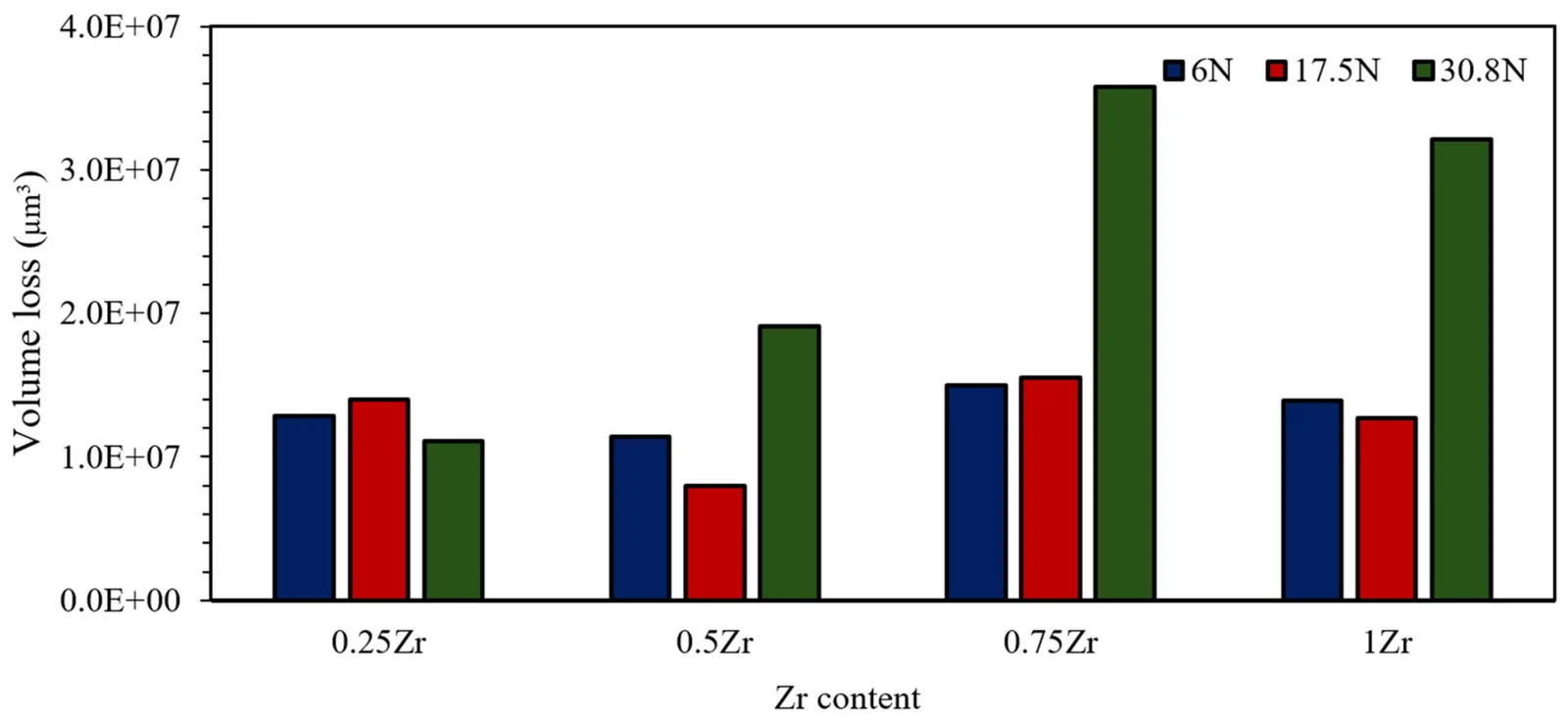
Volume loss of various Mg alloys in PBS under the three normal loads of 6, 17.5, and 30.8 N.

Figure 8 and Figure 9 exhibit the volume losses of different alloys under various normal loads in dry and PBS conditions, respectively. It is expected that Zr would act as a grain refiner in Mg alloys, which can promote the formation of a finer and more homogeneous microstructure. Grain refinement can improve mechanical properties, such as hardness and strength, and it can potentially increase corrosion resistance by reducing microstructural heterogeneities that can induce preferential sites for localised corrosion. As the Zr content increases, the resulting reduction in grain size improves the alloy’s resistance to plastic deformation, and this can then limit the penetration of asperities and abrasive particles into the alloy surface; thereby, volume loss is decreased. The more uniform microstructure promotes the formation of a stable and homogeneous layer, which can provide additional protection against degradation during rubbing. The results of Figure 8 and Figure 9 only confirm these expectations in some testing conditions. It is evident that the influence of Zr content is coupled with the effect of the environment and how much contact pressure is present at the interface.

Under a normal load of 6 N and in a dry environment, the influence of the Zr content does not show a clear trend, in which the volume loss is increased by a change in Zr content from 0.25% to 0.5%, followed by a subtle reduction from 0.5% to 0.75%, and then the volume loss again increases with an increase in the Zr content to 1.00%. Under the same normal load of 6 N but in PBS, this trend is approximately reversed. The increase in Zr content first reduces the volume loss, then an increase is observed, and finally, the volume loss reduces to a lower value. Under a normal load of 17.5 N, the 0.25Zr and 0.5Zr alloys demonstrate the minimal volume losses in dry and PBS environments, respectively. Interstingly, in both environments and under a normal load of 17.5 N, the 0.75Zr alloy showed the maximal volume losses. With a change in normal load to 30.8 N, the 0.5Zr and 0.25Zr alloys showed the minimal volume losses in dry and PBS environments, respectively; however, the 0.75Zr alloy showed the maximal volume loss (similar to the results observed under 17.5 N).

**Influence of normal load and testing environment**

As for the influence of the normal load in the dry condition, it is observed that a clear non-linear trend exists, in which the volume loss increases with an increase in the normal load. This trend is partly consistent with Archard’s law, through which the volume loss increases linearly with the normal load. Overall, the increase in the material volume loss is steeper when changing the normal load from 6 N to 17.5 N compared to the increase observed when changing the normal load from 17.5 N to 30.8 N. The influence of the normal load in the PBS condition is not as straightforward as that in the dry condition. In PBS solution and for all alloys (except 0.25Zr alloy), the samples tested under 30.8 N demonstrate the maximal volume loss, while the difference in the volume loss under 6 N and 17.5 N is subtle and depends on the Zr content. This is in contrast with what is observed in dry conditions, wherein the change in the volume loss is noticeable when changing the normal load from 6 N to 17.5 N. This shows the strong influence of the PBS environment, such that it can reverse the influence of the normal load. This influence is highlighted in the 0.25Zr alloy, in which the volume loss does not change noticeably with the change in the normal load from 6 N to 30.8 N. To summarise the influence of the normal load on the volume loss in dry and PBS conditions, the ratio of the volume loss under 17.5 N and 30.8 N with respect to that under 6 N was calculated and listed in Table 2. One can see that these ratios are higher in the dry environment, and this further highlights that the normal load influences the volume loss much more noticeably in the dry environment. The PBS solution can change this trend depending on the Zr content and how much the contact pressure changes between the test batches. The results of volume loss clearly indicate the influence of the environment within which the test is conducted. The volume losses for each alloy in dry conditions are much higher compared to those in PBS under the same normal load. This influence is much more pronounced in the cases tested under the normal loads of 17.5 N and 30.8 N across various alloys. The PBS environment strongly affects the influence of the normal load in the 0.25Zr alloy, in which the material volume loss does not change remarkably with the normal load.

## 4. Discussion

Magnesium alloys continue to attract interest in biomedical implants because of their biodegradability, favourable stiffness, and ability to reduce stress shielding relative to permanent metallic implants. However, their clinical adoption remains constrained by wear and corrosion. Prior studies have consistently identified the interaction of wear and electrochemical dissolution as a major challenge for temporary load-bearing implants [2,3,7,28]. The present work investigates how the Zr content in a series of new Mg-based alloys (Mg-3Zn-xZr-1Y, x = 0.25, 0.50, 0.75, 1.00 wt.%) influences their tribological response in both dry and PBS conditions across varying contact pressures. The findings demonstrated how the interactions between environmental exposure, alloy composition, and mechanical loading govern the degradation behaviour of the alloys.

Taking into account the volume loss findings through confocal microscopy, the effect of Zr content on the volume loss was found to be highly dependent on both the applied normal load and the testing environment, indicating that the tribological response of the investigated Mg alloys is governed by the interaction of multiple factors rather than by alloy composition alone. Although Zr is widely recognised as a grain refiner that improves hardness, strength, and microstructural homogeneity, the present results demonstrate that these beneficial effects do not translate directly into a monotonic reduction in wear. In the authors’ previous study [25], the same Mg–3Zn–xZr–1Y alloy system was characterised by optical and SEM microscopy, Vickers hardness measurements, and tensile testing. The microstructural observations showed Zr-dependent changes in grain morphology and the distribution of intermetallic phases, consistent with the established grain-refining role of Zr in Mg alloys [31,32]. The previously measured hardness values for the 0.25Zr, 0.50Zr, 0.75Zr, and 1.00Zr alloys were approximately 51.5, 49.2, 50.8, and 48.6 HV, respectively, demonstrating that hardness did not increase monotonically with Zr content. The tensile properties were found to be dependent on the alloy composition; notably, the 0.50Zr alloy exhibited the most favourable tensile behaviour, with an ultimate tensile strength of approximately 201.6 MPa, a yield strength of 80.7 MPa, a Young’s modulus of 40.4 GPa, and an elongation of 11.8% [25].

In the present study, the 0.50Zr alloy exhibited the lowest volume loss under 30.8 N in dry conditions and under 6 N and 17.5 N in PBS. This suggests that its favourable combination of microstructure and mechanical properties may have contributed to improved resistance to material removal during sliding. Similarly, the 0.25Zr alloy, which exhibited the highest hardness among the four compositions, showed the lowest volume loss under 17.5 N in dry conditions and 30.8 N in PBS. Its slightly higher hardness may have improved resistance to abrasive penetration and plastic deformation during sliding. However, the 0.75Zr alloy exhibited the greatest volume loss at both 17.5 and 30.8 N in both environments, despite having the second-highest hardness among the investigated alloys. This demonstrates that hardness alone cannot fully explain the observed wear behaviour, consistent with the findings of Huang et al. [33], who reported that the wear resistance ranking of three Mg alloys under higher applied loads was inversely related to their hardness and yield strength. Beyond bulk hardness, the distribution and morphology of intermetallic phases may also influence the wear resistance of Mg alloys by affecting localised deformation and material detachment during sliding. For example, Chen et al. [34] reported that coarse intermetallic phases in AZ91 Mg alloys promoted localised stress concentrations and crack initiation at the interfaces with the Mg matrix during sliding, contributing to surface material detachment. A similar mechanism may contribute to wear debris generation in the present alloys, depending on the morphology and distribution of their intermetallic phases. Once liberated, relatively hard and angular debris may become trapped between the contacting surfaces and intensify abrasive wear through additional cutting and ploughing [29]. However, repeated contact loading may also cause debris fragmentation, altering particle size and its abrasive action. Furthermore, Pulido-González et al. [35] reported that oxidised wear debris in Mg alloys could agglomerate during repeated sliding, forming a surface layer with protective properties against further wear. Therefore, the observed differences in wear resistance may reflect the combined influence of bulk mechanical properties, microstructural characteristics, and the evolution of wear debris during sliding.

Under several testing conditions, particularly at the higher normal loads of 17.5 N and 30.8 N, the 0.75Zr alloy consistently exhibited the greatest volume loss, whereas the alloys with lower Zr contents (0.25Zr or 0.5Zr) frequently showed superior wear resistance. The poor tribology performance of 0.75Zr alloy may be linked to the previously studied corrosion performance of this alloy in the authors’ previous work [25]. The volume loss observations in this study may suggest that an optimum Zr concentration may exist beyond which further grain refinement no longer provides additional protection against material removal. Instead, excessive Zr additions may alter the microstructure in a manner that could affect deformation behaviour and third-body particle generation. The reversal of Zr-dependent trends between dry and PBS environments further indicates that the influence of alloy composition cannot be considered independently of the surrounding environment, as the effectiveness of microstructural refinement is closely linked to the prevailing damage mechanisms operating during sliding.

The normal load and testing environment exerted a substantially greater influence on volume loss than Zr content alone. It is noted that greater normal load increases real contact area, plastic deformation, and disruption of surface oxides. The normal load sensitivity observed in this study agrees with previous findings for AZ31B and AZ31 alloys where increasing normal load enlarged wear scars and accelerated material removal [21,22]. In the dry sliding of the tested alloys, the increase in volume loss with increasing normal load generally followed the expectations of Archard’s wear law, although the relationship was distinctly non-linear, with a much steeper increase occurring between 6 N and 17.5 N than between 17.5 N and 30.8 N. This behaviour suggests that the wear process becomes progressively less sensitive to further increases in normal load, potentially because of work hardening, the development of compacted wear debris, or the formation of mechanically mixed surface layers that partially reduce the wear rate. In contrast, the PBS environment significantly altered this load dependence, reducing the overall volume loss for all alloys and diminishing the influence of increasing contact pressure, particularly for the 0.25Zr alloy where wear remained nearly unchanged across the entire load range. The markedly lower volume losses in PBS are consistent with the combined lubricating and heat compensating effects of the solution, which reduce direct asperity interactions, minimise frictional heating, and may facilitate the formation of surface films that remain sufficiently adherent during sliding. Consequently, the results demonstrate that wear performance is governed by a tribocorrosion response rather than by purely mechanical wear, with the surrounding environment playing a dominant role in determining the extent to which applied load influences material removal.

Through observation in the SEM images of the Mg alloy samples tested under a normal force of 30.8 N in dry conditions (Figure 3), abrasive wear was found to be the dominant degradation mechanism, primarily through ploughing and localised cutting accompanied by limited material delamination. This is consistent with wear mechanisms previously reported for other magnesium-based alloys [36,37]. Also, when comparing the SEM images of the alloys with the lowest and highest Zr contents under a normal force of 30.8 N in dry conditions, increasing the Zr content from 0.25 to 1.00 wt.% (Alloy 1 and Alloy 4, respectively) enhanced the resistance of the alloy surface to plastic deformation and produced a more uniform worn-surface morphology, which may indicate improved wear resistance in Alloy 4 (Figure 8). 

Focusing on the SEM images of Alloys 1 and 4 tested under a normal force of 30.8 N in PBS conditions (Figure 4), the wear morphology showed shallower scratches with less localised cutting when compared to the same alloys tested in the dry conditions. This reduction may be partly attributed to the lubricating effect of the PBS solution, which can reduce direct contact between the asperities of the mating surfaces. This is consistent with the lubricating effect of saline solution reported in another study [38]. Also, this reduction could be attributed to the moderate cutting action of detached wear debris. In the Mg alloy samples tested in the PBS solution, the presence of PBS promoted oxidation and electrochemical reactions on the fresh alloy surface, resulting in the formation of the tribologically modified layer. The local cracking, fragmentation, and delamination of this layer indicated that it was formed and removed during sliding. Therefore, the dominant degradation mechanism changed from predominantly abrasive wear under dry conditions to a combination of moderated abrasive wear and oxidation/corrosion in PBS. This combined mechanical and electrochemical degradation mechanism is the key characteristic of tribocorrosion [9,17].

The EDS results presented in Figure 5 further supported the occurrence of tribocorrosion under PBS conditions involving oxidative surface degradation in addition to abrasive wear. The marked compositional difference between these regions indicates that tribocorrosion occurred non-uniformly across the wear scar. The oxygen-rich tribologically modified layer likely formed through the accumulation and compaction of oxidised wear debris and corrosion products, whereas continuous abrasion within the smoother grooves limited their accumulation and exposed the underlying Mg-rich alloy.

There are a number of limitations associated with this study. First, the investigated Mg alloys were limited to four zirconium concentrations, which may not fully capture the influence of Zr content across a broader compositional range. In addition, the tribological experiments were conducted under a limited set of testing conditions, including the normal load, sliding stroke, sliding velocity, and reciprocating frequency. Furthermore, the tribology experiments were performed in PBS, which does not fully replicate the complexity of the physiological environment, such as the presence of proteins, cellular activity, or long-term degradation processes, all of which can influence the degradation and tribology behaviour of biodegradable Mg alloys. Consequently, the findings are specific to the tested conditions and may not be extended to situations beyond the tested parameter ranges or alloy compositions. In the present study, the primary focus was on material loss and surface degradation mechanisms; therefore, the coefficient of friction (COF) was not monitored. COF measurements could provide complementary insight into the tribological response and are suggested to be considered in future investigations. Also, this study focused on material volume loss as the primary measure of tribocorrosion performance and did not include electrochemical measurements such as corrosion potential and/or current evolution, or the calculation of wear rates [39]. As another limitation in the present study, the tribological tests were repeated three times for a selected number of samples to assess the repeatability of the measurements. Similar to the authors’ previous studies [29,30], the results of these repeat tests showed very good agreement in the estimated volume loss, providing confidence in the repeatability and reliability of the volume loss measurements. Thus, the presented findings are exploratory and should be interpreted cautiously. Electrochemical measurements of active alloys, such as the Mg alloys used in this study, are more challenging than those of passive metals [40] and therefore warrant further investigation. As another possible direction for future research, approaches to enhance corrosion resistance through surface modifications, such as coatings and thermal treatments, could also be investigated [41,42]. Future work can also include testing under distilled water conditions to distinguish the effects of liquid lubrication from those associated with the chemical and electrochemical characteristics of PBS. This will provide further insight into the environmental effects on the wear and tribocorrosion behaviour of the developed alloys.

## 5. Conclusions

This study investigated the tribological behaviour of Mg–3Zn–xZr–1Y alloys containing 0.25–1.00 wt% Zr under dry and PBS conditions using a customised ball-on-disc tribometer. Abrasive wear, characterised primarily by ploughing and localised cutting, was identified as the dominant degradation mechanism under dry conditions. In PBS conditions, the dominant degradation mechanism was found to change from predominantly abrasive wear under dry conditions to a combination of moderated abrasive wear and oxidation/corrosion. Sliding in PBS consistently resulted in lower volume loss when compared to dry sliding in all the tested conditions. This indicates that the lubricating effect of the electrolyte and the formation of surface films can outweigh corrosion-assisted material degradation. The effect of Zr content on the volume loss of the developed Mg alloys was found to be highly dependent on both the normal load and the testing environment. In both the dry and PBS conditions under the higher normal loads of 17.5 N and 30.8 N, the 0.75Zr alloy showed the maximum material loss, whereas the alloys with lower Zr contents (0.25Zr or 0.5Zr) exhibited better wear resistance. The results of this study provide insights for the design and fabrication of biodegradable magnesium alloys for load-bearing biomedical implant applications and establish a foundation for future studies under more physiologically representative conditions.

## Figures and Tables

**Table 1:** Experimental materials and testing parameters for the tribology tests conducted in this study.

Alloy	Nominal Chemical Composition (wt%)	Testing Medium	Normal Load (N)	Maximum Hertzian Contact Pressure (MPa)	Sliding (mm)	Frequency (Hz)	Number of Cycles
Alloy 1	Mg–3Zn–0.25Zr–1Y	Dry	6, 17.5, 30.8	421, 601, 735	2	1	1800
Alloy 2	Mg–3Zn–0.50Zr–1Y	Dry	6, 17.5, 30.8	421, 601, 735	2	1	1800
Alloy 3	Mg–3Zn–0.75Zr–1Y	Dry	6, 17.5, 30.8	421, 601, 735	2	1	1800
Alloy 4	Mg–3Zn–1Zr–1Y	Dry	6, 17.5, 30.8	421, 601, 735	2	1	1800
Alloy 1	Mg–3Zn–0.25Zr–1Y	PBS	6, 17.5, 30.8	421, 601, 735	2	1	1800
Alloy 2	Mg–3Zn–0.50Zr–1Y	PBS	6, 17.5, 30.8	421, 601, 735	2	1	1800
Alloy 3	Mg–3Zn–0.75Zr–1Y	PBS	6, 17.5, 30.8	421, 601, 735	2	1	1800
Alloy 4	Mg–3Zn–1Zr–1Y	PBS	6, 17.5, 30.8	421, 601, 735	2	1	1800

**Table 2:** The volume loss ratio of various alloys in dry and PBS environments. The volume losses of the normal loads of 17.5 N and 30.8 N are compared with those under 6 N in each environment.

**Dry Environment**				
**Force Ratio**	**0.25Zr**	**0.50Zr**	**0.75Zr**	**1.00Zr**
30.8: 6	8.05	5.63	8.02	5.59
17.5: 6	2.93	3.67	5.40	3.53
**PBS ** **Environment**				
**Force Ratio**	**0.25Zr**	**0.50Zr**	**0.75Zr**	**1.00Zr**
30.8: 6	0.868	1.67	2.39	2.31
17.5: 6	1.09	0.701	1.04	0.913

## Data Availability

The original contributions presented in this study are included in the article. Further inquiries can be directed to the corresponding author.
